# Perceptual Learning via Modification of Cortical Top-Down Signals

**DOI:** 10.1371/journal.pcbi.0030165

**Published:** 2007-08-17

**Authors:** Roland Schäfer, Eleni Vasilaki, Walter Senn

**Affiliations:** Department of Physiology, University of Bern, Bern, Switzerland; University College London, United Kingdom

## Abstract

The primary visual cortex (V1) is pre-wired to facilitate the extraction of behaviorally important visual features. Collinear edge detectors in V1, for instance, mutually enhance each other to improve the perception of lines against a noisy background. The same pre-wiring that facilitates line extraction, however, is detrimental when subjects have to discriminate the brightness of different line segments. How is it possible to improve in one task by unsupervised practicing, without getting worse in the other task? The classical view of perceptual learning is that practicing modulates the feedforward input stream through synaptic modifications onto or within V1. However, any rewiring of V1 would deteriorate other perceptual abilities different from the trained one. We propose a general neuronal model showing that perceptual learning can modulate top-down input to V1 in a task-specific way while feedforward and lateral pathways remain intact. Consistent with biological data, the model explains how context-dependent brightness discrimination is improved by a top-down recruitment of recurrent inhibition and a top-down induced increase of the neuronal gain within V1. Both the top-down modulation of inhibition and of neuronal gain are suggested to be universal features of cortical microcircuits which enable perceptual learning.

## Author Summary

Perceptual learning improves sensory stimulus discrimination by repeated practicing. The improved stimulus discrimination is often thought to arise either from modified stimulus representation in the sensory cortex, or from modified readout from the sensory cortex by higher cortical units. Both explanations, the modified sensory representation and the modified readout, have their advantages and disadvantages. Modifying the stimulus representation within the early sensory cortex may lead to an improvement on one discrimination task, but may have long-lasting negative effects on another task. Modifying the task-specific readout by a higher cortical area, on the other hand, prevents this undesirable interplay between tasks. However, it may be difficult for the readout units to compensate for stimulus distortions produced by interactions in the sensory cortex. Here we show that top-down modulation of the early stimulus representation combines the benefits of task specificity and of eliminating inherent distortions. The task specificity naturally arises from distinct task representations in the higher cortical areas which, by top-down signaling, reversibly improve the task-related representation of sensory stimuli. Based on a visual brightness discrimination task, we show that modifying top-down projections alone can explain psychophysical and electrophysiological data on perceptual learning.

## Introduction

Since Plato's *Allegory of the Cave* (360 BC) and Kant's *Critique of Pure Reason* (1787), it is often suggested that our perception of objects in the outer world can never tell us what they really are. “If men had green glasses in place of their eyes, they would perceive the objects as green, and never be able to tell whether this color was intrinsic to the objects or just of our perception” (letter of Heinrich von Kleist to his fiancée Wilhelmine von Zengen, 22 March 1801, in which he describes Kant's ideas, http://www.kleist.org [in German]). In a contemporary neuroscientific version of the empiricist's position, one may argue that the perception of visual objects is always distorted by the nonlinearities in the visual pathway, and in particular by the intrinsic circuitry of primary visual cortex (V1). In fact, any visual input is filtered by the neuronal processing in V1 before reaching consciousness. For instance, collinear edges are enhanced by the intrinsic V1 circuitry [1], and our brightness perception will never match the physical luminance. Nevertheless, perceptual training without teacher feedback may still improve our brightness discrimination abilities [1], casting certain doubts about the strict empirical view. How then is it possible to reach more veridical perceptions by just “pure reason,” i.e., by intrinsically adapting the cortical dynamics without being told about the mismatch between percept and true physical quality?

We show in a model that top-down modulation of V1 during unsupervised perceptual learning can suppress intrinsic nonlinearities in V1. The top-down suppression leads to a faithful neuronal representation of the sensory input. The underlying neuronal mechanisms are elaborated in an example of brightness discrimination. In this example, a flanking light bar which is closely aligned in prolongation of a test bar acts as a visual context. This flanking bar biases the brightness perception of the test bar. In the presence of the flanking bar, the test bar is perceived to be brighter than it actually is. Clearly, this enhanced brightness perception is helpful when extracting collinear line elements against some noisy background [2,3]. However, when the task consists of comparing the brightness of the test bar with a displaced single reference bar, then the collinear flank distorts the brightness comparison [4]. The brightness of the test bar is overestimated because the underlying neuronal population representing the test bar within V1 is recurrently excited by the corresponding population representing the collinear flanking bar [1]. We show that top-down input can remove this contextual bias by activating recurrent inhibition within V1. The recurrent inhibition cancels the lateral excitation and linearizes the brightness representation of the test bar, allowing for a faithful perception. An additional top-down induced gain increase in V1 further enhances the sensitivity to brightness differences.

Perceptual learning, i.e., the change of perception following sensory experiences, is typically explained as a modification of either the feed-forward synaptic pathway to V1 [5–7], or recurrent connections within V1 [8–11] triggered by repeated practicing. Because these synaptic modifications would affect any input stream through V1, however, perceptual learning would inevitably deteriorate the information processing in other situations. Although negative transfer of learning to other tasks is known to appear (see, e.g., [12]), perceptual learning is typically task-specific and does not deteriorate perception in other tasks; see, e.g., the reviews [13,14]. While improving in brightness discrimination between a context-modulated test bar and a displaced reference bar, for instance, the edge detection capability is expected to not suffer. In fact, the mutual enhancement of collinear light bars is advantageous for extracting lines in a noisy scene, as required for contour integration in everyday scenes [4]. Hence, models of perceptual learning have to explain how improvement on one task is possible without interference with others. An intriguing possibility is that perceptual learning might be based on modifying a task-dependent top-down input to sensory areas, as opposed to a permanent change of the bottom-up input stream [15–17]. Taking up this idea we show how top-down signaling from a higher cortical area to V1 could modify the neuronal processing in this lower area, consistent with both electrophysiological recordings in V1 and psychophysical experiments on perceptual learning.

## Results

### Facilitation by Collinear Flanks

**Figure 1:**
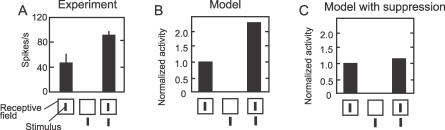
Contextual Modulation of Neuronal V1 Responses (A) Extracellular recordings from an orientation selective neuron in V1 of a monkey, activated by an optimal bar within its receptive field. The neuron does not respond if the bar is outside the receptive field. However, the neuronal response is strongly facilitated if the two collinear bars are presented together (left to right, Figure adapted from [18]). (B) The neuronal responses are reproduced by a network of two recurrently connected linear threshold units (see Equation 1 and Figure 2A). (C) The same model neuron in the presence of focal attention, modeled by top-down activation of recurrent inhibition (see text and Figure 2B1) is barely affected by the contextual bar (the parameters were chosen to reproduce the behavioral learning curves presented in the following, and the facilitation is therefore not fully suppressed). The suppression of the neuronal facilitation in the model is at least partially confirmed by in vivo recordings in monkeys during focal attention (see [18] and main text). Parameter values: g = 1, θ = 27 Hz, w_ij_ = 0.55, k = 0.45, w_0_ = 8.3, τ_D_ = 0.04s, θ_inh_ = 120 Hz.

We first modeled the in vivo experiment which reveals a nonlinear facilitation of V1 neurons triggered by collinear flankers in their extra-classical receptive field (see [18] and Figure 1). According to this experiment, the response of a V1 neuron to a light bar in its receptive field is stronger when an additional collinear bar is present nearby. This second flanking bar alone does not evoke any response as it is outside the receptive field. However, if it appears together with the one within the receptive field, the response is almost doubled (Figure 1A).

**Figure 2:**
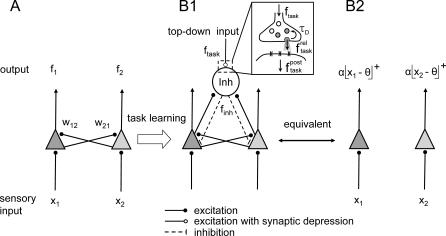
Top-Down Suppression of Recurrent Excitation in V1 (A) Mutually exciting neurons in V1, each responsive to a bar within their corresponding receptive field, determine the neuronal responses shown in Figure 1B and described by Equation 1. (B1) Recurrent inhibition within V1 (f_inh_), which is silent in the absence of top-down input, can be activated by an input from a higher cortical area (f_task_). Inset: the top-down input undergoes short-term synaptic depression which normalizes the drive to the inhibitory neuron: if the presynaptic firing rate, f_task_, is above some critical frequency (of roughly 25 Hz), the vesicle release rate, 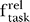 , and the effective postsynaptic rate, 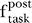 (which is the product of 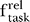 times the synaptic strength), both saturate. The synaptic strength can be tuned such that the saturation value of 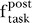 just overcomes the firing threshold, making inhibition linear in the inputs from the two pyramidal neurons (Equations 2 and 3). (B2) As a consequence, any strong top-down input to the inhibitory neuron leads to a cancellation of the mutual excitation among the pyramidal neurons through their own drive of the recurrent inhibition. The circuitry in B1 then becomes equivalent to a decoupled V1 circuitry which feeds sensory input through, as the recurrent connections would not exist (compare Equations 4 and 5).

The same nonlinear response properties are also present in the model neuron (Figure 1B). This receives direct input from the stimulus within its receptive field, but only indirect input from the collinear flank outside the receptive field (Figure 2A). The indirect, lateral input by itself may only drive the neuron towards firing threshold, not above. Together with the direct, supra-threshold input, however, it visibly adds to the response of the model neuron.

Because the full model network is recurrent and includes a positive feedback loop (Figure 2A), a quantitative description of the different neuronal responses requires some formal treatment. A minimal network model consists of two mutually excitatory neuronal populations, the first of which is directly driven by the test bar, and the second by the flanking bar. To reveal the main idea, we identify each of these populations with a single prototypical neuron reflecting the dynamics of the whole population. In a further simplification, the firing rates of these two prototypical neurons representing the test and flanking bar (f_1_ and f_2_, respectively) are considered to be threshold-linear functions of the total synaptic inputs,

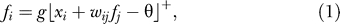
where g(=1) is the neuronal gain, x_i_ the feedforward input of the corresponding light bar, w_ij_ the lateral synaptic strength from neuron j to neuron i (with i ≠ j), and θ the firing threshold. The brackets [z]^+^ = max(0,z) denote the identity function with cut-off at 0. Although the full model takes account of the neuronal dynamics (see Materials and Methods), the steady-state considerations presented here and below are enough to understand the results.

When stimulus 1 (test bar) is presented alone, say with input strength x_1_ = 2θ, neuron 1 will respond alone with strength f_1_ = θ, provided that the synaptic strength from neuron 1 to neuron 2 (w_21_) is not too strong. In turn, if only stimulus 2 (flanking bar) is presented, neuron 1 will not respond because the second neuron (which then fires with f_2_ = θ) will not drive neuron 1 above threshold (say that w_12_ = w_21_ ≈ 0.5). However, when both stimuli are present (x_1_ = x_2_ = 2θ), the two neurons mutually excite each other and their firing rate is roughly doubled (f_i_ = g(x_i_ − θ)/(1 − gw_ij_) ≈ 2θ, for both i = 1,2). This formally explains the strong response increase to two collinear light bars extending across and beyond the receptive field (Figure 1A and 1B).

### Suppression of Intrinsic V1 Circuitry

Although the mutual excitation between the collinear edge detectors is beneficial for extracting lines, it may be detrimental for other tasks such as brightness discrimination. How is it possible to suppress the perceptual distortions imposed by the recurrent V1 circuitry? Cutting off or permanently modifying the lateral connections through learning is not a solution since otherwise the facilitation effect would be lost when needed to extract line segments in a noisy surrounding. However, it is possible to compensate for the lateral excitation in V1 by a top-down recruitment of inhibition. Different wirings are conceivable which yield a cancellation of the lateral excitation. Recurrently connected feedback inhibition through tightly coupled inhibitory networks is a characteristic cortical architecture [19–21], and it is particularly challenging to test whether such a circuitry may also serve for suppressing the facilitation. In fact, by driving recurrent inhibition within V1, each neuron can be inhibited by approximately the same amount as it is facilitated by its surrounding excitatory neurons. Since this recurrent inhibition is enabled by the top-down input, the suppression can transiently be turned on when required by the task, without leaving long-lasting modifications of the intrinsic V1 circuitry.

To test these ideas, we considered a population of inhibitory neurons driven by both excitatory neuronal populations within V1 and some task population in a higher cortical area. Again, each population is identified by a prototypical neuron. We assume that the firing rate of the inhibitory neuron is a threshold-linear function of the total input with gain one,

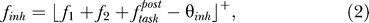
where f_1_ + f_2_ is the total firing rate of the V1 neurons representing the test bar and the flank, 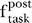 , is the effective postsynaptic rate at the synapse projecting from the task neuron to the inhibitory neuron, and θ_inh_ is the firing threshold for inhibition (Figure 2B1). We further assume that the top-down synapses undergo short-term synaptic depression such that the effective postsynaptic current becomes the same for any strong presynaptic firing rate [22,23]. Whenever the top-down firing rate from the task neuron is turned on, say with f_task_ > 25 Hz, then the effective postsynaptic rate 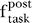 reaches some saturation value. We chose the strength of synaptic depression such that this saturating rate roughly cancels the firing threshold, 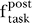 ≈ θ_inh_. Due to saturation, this approximate equality remains true even when the top-down firing rate f_task_ is strengthened after perceptual learning. Hence, when the top-down drive is strong, the firing rate of the inhibitory neuron (Equation 2) gets linearized,

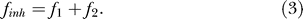



If the inhibitory neuron recurrently projects to the two excitatory neurons with strength k, the latter receive the additional inhibitory current −kf_inh_ = −k(f_1_ + f_2_). Adding this to the total postsynaptic current of the excitatory V1 neurons (Equation 1) leads to the recurrent firing rate equations

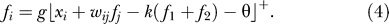



Next we assume that the strength of the inhibitory and the recurrent excitatory synapses are roughly equal in absolute strength, k = w_ij_. The additional drive from the collinear flank is then canceled by the recurrent inhibition, simplifying Equation 4 to f_i_ = g [x_i_−kf_i_−θ]^+^. Solving this equation for the postsynaptic firing rates f_i_ then yields

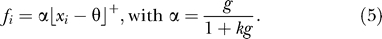



The reasoning shows that the response of each excitatory V1 neuron in the presence of strong top-down input is approximately a threshold-linear function of the feed-forward input, independent of the firing rate of the other neurons (Equation 5). The recruitment of recurrent inhibition virtually breaks up the excitatory recurrent circuitry (symbolized by Figure 2B2). As a consequence, a particular V1 output neuron in the presence of strong top-down input will only respond to a light bar in its classical receptive field, and it is only marginally affected by an additional light bar in its non-classical surround (Figure 1C). This suppression of the surround modulation induced by focal attention is partially confirmed by single cell recordings in monkeys (it was confirmed to be the case for two monkeys before learning, and it became pronounced for one of the two monkeys after learning, see [18]).

### Top-Down Gain Modulation of Excitatory Neurons

Before being ready to explain the perceptual learning results, we need to introduce an additional feature to our model network. Unsupervised training and focal attention has been shown to improve brightness discrimination in two specific ways: (i) the bias imposed by a collinear light bar is suppressed and (ii) the sensitivity to brightness discrimination is enhanced [1,4]. While bias suppression could be explained by the suppression of the recurrent feedback (Equations 1 to 5), the sensitivity enhancement could be explained by an additional gain increase of V1 neurons.

One candidate neuron for a top-down induced gain increase would be the layer 2/3 (L2/3) pyramidal neurons within V1, the “input neurons” in our model network. However, a gain increase in these neurons would roughly be canceled by the simultaneous suppression through recurrent inhibition we are postulating. In fact, a look at Equation 5 shows that the gain of the local V1 microcircuitry in the presence of the top-down suppression, α = g/(1 + kg), saturates quickly when increasing the gain g of the neuronal transfer function. To at least overcome the effective reduction of the circuitry gain when recruiting inhibition (acting through k), we still assume that the top-down input to the L2/3 pyramidal neurons increases their gain g. This gain increase is modeled by a nonlinearly increasing and saturating function of the top-down frequency f_task_, similarly to the one measured in vitro [24]. For instance, a top-down activated gain increase from g^0^ = 1 to g^1^ = 2 keeps the original network gain α constant when co-activated with recurrent inhibition (α^0^ = g^0^ = 1 and α^1^ = g^1^/(1 + kg^1^) = 1 with k = 0.5).

**Figure 3:**
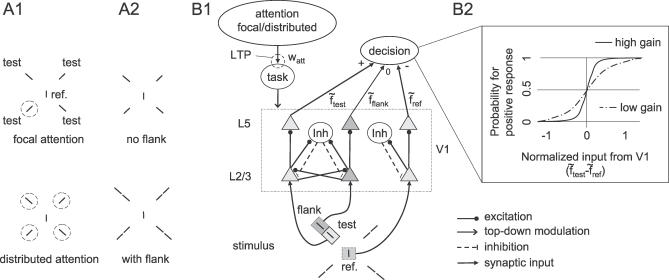
Brightness Discrimination Task and Model (A) The stimulus in the brightness discrimination task consists of four test bars surrounding a reference bar, with one of the four test bars deviating in brightness from the others. The task is to report whether this deviating test bar is brighter than the reference bar or not (see [1,18]). Preceding cues may either focus attention to that deviating test bar or distribute attention across all four test bars (A1). To study contextual interactions in both attentional states, the test stimuli were presented with or without a collinear flanking bar (A2). (B1) The model V1 consists of layer 2/3 (L2/3) pyramidal neurons which are recurrently connected and receive feedforward sensory input. The L2/3 neurons feed through corresponding layer 5 (L5) pyramidal neurons to a decision network in a higher cortical area. The two L2/3 neurons responsive to the nearby *flank*- and *test*-bar, respectively, are recurrently connected and drive a common inhibitory neuron feeding back to them. The spatially displaced reference bar (*ref*) drives a separate L2/3 neuron with its own recurrent inhibition. Top-down input depolarizes the inhibitory neurons through depressing synapses (as explained in Figure 2) and additionally increases the gain of L2/3 and L5 pyramidal neurons. This attentional drive is weak for distributed and strong for focal attention. Learning consists in further strengthening the top-down synapses (w_att_) from the attentional centers to the task neuron, modeled by Hebbian LTP. (B2) The decision population is stochastically activated (1 or 0), with an activation probability being a sigmoidal function of the difference between the V1 output encoding the test and reference bar, fx̃_test_ − fx̃_ref_. The top-down gain increase steepens this probabilistic decision function (solid line: task neuron active; dash-dot line: not active).

To nevertheless achieve a net gain increase of the whole network, we assume that L2/3 neurons feed through layer 5 (L5) pyramidal neurons before projecting to higher cortical areas (see, e.g., [25,26]). We incorporate a top-down gain increase also in these L5 pyramidal neurons (Figure 3A1). Consistent with the experimental findings [24], the gain of the L5 pyramidal neurons, denoted by gx̃, is again modeled by a monotonically increasing and saturating function of the top-down firing rate f_task_. The overall circuitry gain α then has the form

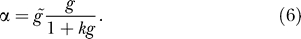



While for top-down input f_task_ < 8.5 Hz we have a gain gx̃ = 1 of the L5 pyramidal neuron, we get an additional gain factor gx̃ = 1.5 for f_task_ = 10 Hz and gx̃ ≈ 3.3 for f_task_ = 45 Hz, for instance.

### Brightness Discrimination Task and Model Network

The top-down recruitment of inhibition and the top-down gain increase are the two key elements which explain perceptual learning in the brightness discrimination task considered in [1,18]. In this task, a subject (human or monkey) has to judge whether one of four randomly chosen test bars is brighter or dimmer than a reference bar (Figure 3A). A preceding cue indicates whether the subject has to attend to one (focal attention) or all four test bar locations simultaneously (distributed attention, Figure 3A1). To investigate the effect of collinear light bars onto brightness perception, collinear flanks were placed outside each of the four test bars in half of the stimulus presentations (Figure 3A2). No feedback on the correctness of the brightness decision was given, neither in the experiment nor in the model.

The model architecture consists of three V1 pyramidal neurons in L2/3 (again each representing a population of those) with receptive fields at the position of the relevant test bar, the flanking bar, and the reference bar, respectively (Figure 3B1). The neurons responding to the test and flanking bar are recurrently connected through direct excitation and shared inhibition, while the neuron responding to the reference bar is indirectly inhibited only by its own drive. Attention acts through a task population in a higher cortical area which itself modulates the gain of the L2/3 and L5 pyramidal neurons and drives the inhibitory neurons within V1 towards firing thresholds. As compared with the top-down induced gain increase, the top-down drive of the inhibitory neuron is assumed to saturate earlier by means of synaptic depression (see inset of Figure 2B1).

The decision about the brightness difference between test and reference bar is modeled as a stochastic function of the difference between the L5 output activities fx̃_test_ − fx̃_ref_, as it can be implemented with a classical decision making network [27]: the more fx̃_test_ exceeds fx̃_ref_, the more likely will the test bar be judged to be brighter than the reference bar (see Figure 3B2). We assume that the comprehension of the task by the subject implies the selection of an appropriate decision network in a higher cortical area. This decision network combines the potentially distorted, but relevant, inputs from the lower area, fx̃_test_ and fx̃_ref_, while suppressing the irrelevant input from the flank neuron, fx̃_flank_. We assume that without external feedback about the outcome of the decisions, these bottom-up connections to the decision network are not modified.

### Perceptual Learning by Strengthening Top-Down Input

**Figure 4:**
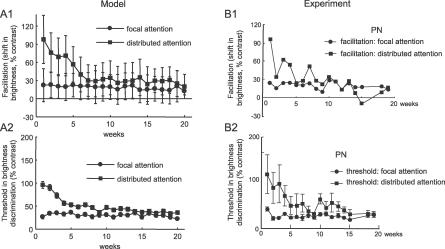
Learning Curves from the Model (A) and the Experiment (B), Reproduced from [1] Training (600 trials per week) reduces the facilitation in brightness perception by a flanking bar (A1, B1) and reduces the threshold in brightness discrimination (A2, B2). In the model, the learning progress is achieved through LTP at synapses from the attentional centers to the task neuron (cf. Figure 3). Increased drive of the task neuron in turn activates the inhibitory neurons and increases the gain of the V1 pyramidal neurons. This explains the reduction in the brightness facilitation and in the discrimination threshold, respectively. Experimental data (adapted from [1]) are from one human subject. Error bars here and in the following figures indicate the standard error of the mean (not shown in B1).

Prior to brightness discrimination training, the top-down input in the case of distributed attention is too weak to activate inhibition within V1. The top-down drive is therefore also too weak to suppress the recurrent excitation between the test and the flanking bar (cf. Equation 1). Due to the unbroken recurrent excitation, the flanking bar enhances the V1 activity and shifts the brightness perception of the test bar towards higher values (facilitation, see also Figure 1B). This brightness shift implies a bias in brightness discrimination in favor of the test bar as compared with the reference bar (Figures 4A1 and 5A1, before learning).

Perceptual learning in our model consists of increasing the drive from attentional centers to the task population through Hebbian modification of the synaptic strength w_att_ (Figure 3B1). Because in the case of distributed attention the attentional input is only weak, say 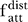 = 16 Hz, and because we assume that before learning the synaptic strength is weak as well, 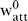 = 0.5, the task neuron is barely activated, 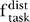 = 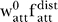 ≈ 8 Hz. During training, long-term potentiation (LTP) of the attention-to-task synapse (w_att_, Figure 3B1) steadily increases towards 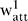 = 1.0. At the lower area, the increasing firing rate of the task neuron then drives the inhibitory neuron towards threshold (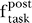 → θ_inh_, see Equation 2). As a consequence, the intrinsic V1 circuitry is suppressed (cf. Equation 5) and the perceptual bias is reduced (Figures 4A1 and 5A1).

**Figure 5:**
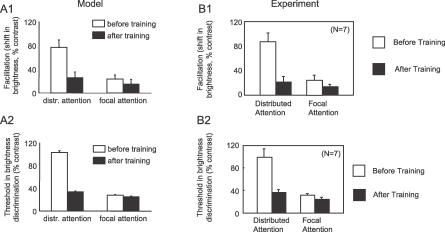
Summary of the Learning Performance for the Model (A) and the Experiment (B) (A) Unsupervised perceptual learning during distributed attention reduces the brightness facilitation (i.e., the decision bias, A1 left) and the discrimination threshold (A2 left) in the model. Focal attention by itself reduces facilitation (A1 right) and threshold (A2 right) before learning, but these quantities are only marginally reduced by the perceptual training. (B) Corresponding experimental data showing the close fit by the model. The figures are adapted from [1]. The number of stimulus presentations and trials in the model correspond to numbers in the experiment (see Materials and Methods). In the model, the average data from the first week (before learning) and the 20th week (after learning) was gathered from seven different runs. In the experiment, data was pooled from five human and two monkey subjects.

Simultaneously to the recruitment of inhibition, the training-based increase of the top-down input during distributed attention, 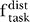 , leads to a gain increase of the L2/3 pyramidal neurons from g^0^ = 1.0 to g^1^ ≈ 2 and in the layer 5 pyramidal neurons from gx̃^0^ = 1.0 to gx̃^1^ ≈ 3 (cf. Equation 6). This gain increase causes the threshold in brightness discrimination to drop (Figures 4A2 and 5A2). Both the reduced brightness facilitation and the reduced discrimination threshold in the case of distributed attention closely reproduce the experimental observations (Figures 4B and 5B).

In the case of focal attention, the facilitation and discrimination threshold are already reduced before learning and do not substantially decrease further during the learning process (Figures 4B and 5B). In our model, this arises because focal attention drives the task neuron considerably above the critical frequency for synaptic depression and also above the gain modulation threshold, even before learning (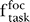 = 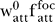 ≈ 24 Hz). As a consequence, inhibition and gain increase are present right from the beginning, reflecting the corresponding high performance in brightness discrimination. The performance does not further improve during learning due to saturation effects. Because synaptic depression limits the drive of the inhibitory neuron, the bias in brightness discrimination is not further reduced. Similarly, because the gain increase saturates with strong top-down input, the discrimination threshold does not further decrease, in full agreement with the psychophysical data (Figures 4 and 5).

### Learning with External Feedback: A Prediction

While in our model the unsupervised learning is purely top-down driven, an external feedback may additionally modulate bottom-up pathways to the decision network in the higher cortical area. Assuming that the decision circuitry for distributed and focal attention is the same also for learning with feedback, we would expect interferences between the modifications of the bottom-up and top-down pathways. Since subjects are not aware of their progress during learning [1], it is in fact likely that the same readout circuitry is used for distributed and focal attention.

An interference induced by the plasticity in the bottom-up and top-down pathways is indeed observed in the model. The teacher feedback is used to modify the synaptic strengths of all three types of L5 inputs to the decision network, fx̃_test_, fx̃_flank_, and fx̃_ref_ (Figure 3B1). We apply a specific form of reinforcement learning to these synapses, an error-correcting learning rule which changes the synaptic strengths only when a wrong decision occurs. Upon missing reward, the synapses are modified in an anti-Hebbian way: if the postsynaptic neuron was erroneously active, the activated synapses weaken, and if the postsynaptic neuron was erroneously silent, the activated synapses strengthen. These correction steps enhance the chance that with the next presentation of the same stimulus the decision network will correctly respond—as far as this is possible in the presence of the brightness distortion imposed by the intrinsic V1 circuitry.

**Figure 6:**
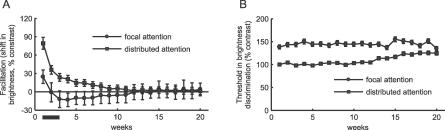
Experimental Predictions (A) Perceptual training with external feedback (starting after the first week) may separate bottom-up and top-down learning phases. The initial decrease in facilitation during the first week (bar from week 1 to 3) represents the fast learning progress based on the changes of the bottom-up connections from V1 to the decision network (see Figure 3A1). While trying to simultaneously maximize the performance for distributed and focal attention, facilitation will be overcompensated for focal attention (negative facilitation during the first weeks). This overcompensation arises when the time scales of the bottom-up and top-down learning are sufficiently different, and if the the stimulus presentations with distributed attention is more frequent than with focal attention (here, 85% distributed and 15% focal attention). In any case, the facilitation for both attentional states eventually disappears due to the slow top-down suppression of the lateral connections (from week 12 onward). (B) Sustained activation of GABA receptors by a GABA agonist disables the top-down induced gain increase in the pyramidal neurons (see [42]), modeled by fixing a unit gain of the L2/3 and L5 neurons, g = gx̃ = 1. As a consequence, the discrimination threshold remains high throughout the perceptual training without feedback (in contrast to the threshold reduction in Figure 4A2). The discrimination threshold for focal attention is even higher than for distributed attention because focal attention still drives the inhibitory neuron, and this reduces the network gain (positive k in Equation 5), without increasing the gain g and gx̃ of the pyramidal neurons.

Simulations show that the facilitation bias is rapidly reduced in the initial phase (Figure 6A). This early progress is enabled by the fast learning of the feed-forward synapses onto the decision network, as compared with the slow learning of the attention-to-task synapses considered before. Because distributed and focal attention are randomly interleaved, the synaptic strengths on the decision network converge to an average between the optimal strength for distributed and focal attention. To compensate for the intrinsic network bias in V1, the fast feed-forward learning causes the facilitation to undershoot in the presence of focal attention, while staying positive in the presence of distributed attention (Figure 6A, up to 11 weeks). The simultaneous top-down learning eventually leads to a suppression of the perceptional bias, and facilitation slowly vanishes for both attentional states (in contrast to the learning scenario without external feedback, see Figure 4A1 and Figure 4B1).

## Discussion

We considered a model of perceptual learning which is based on modifying top-down rather than bottom-up (see, e.g., [5]) or intrinsic V1 connections [8–10]. In the context of a brightness discrimination task, the top-down input (i) suppresses recurrent excitation within V1 and (ii) enhances the gain of the pyramidal neurons. The top-down input linearizes the input–output transfer function of V1 by recruiting recurrent inhibition which in turn cancels the mutual excitation between collinear edge detectors. While this suppression of the intrinsic V1 nonlinearities reduces the facilitation bias, the sensitivity to brightness differences is enhanced by increasing the gain of the V1 pyramidal neurons. Both mechanisms could be related to the specific organization of sensory cortices with an information stream passing first through L2/3 and then through L5 (see, e.g., [26]). The top-down suppression of lateral excitation among L2/3 pyramidal cells may be mediated by an electrically coupled population of inhibitory neurons in a higher cortical layer [19,22]. A top-down gain increase of neurons in lower visual areas is observed during attentional modulations [28]. In our model, the top-down gain increase is crucially required in L5 pyramidal neurons and could be achieved through calcium spikes in the distal dendrites of these neurons, elicited by the joint top-down and bottom-up input [24].

### Feedback versus Feed-Forward and Lateral Models

The top-down model of perceptual learning has several advantages over models which either change the lateral connections within the sensory area, or which change the feed-forward (bottom-up) connections to a read-out population. First, models which intrinsically change the early stimulus representation [8–11] can explain perceptual learning only at the expense of a degradation on other tasks. A task-specific top-down input, instead, can specifically suppress or enhance a certain pre-wiring without interference with other tasks.

Second, models which explain perceptual learning by only adapting the read-out connections to a higher cortical area [5,6,17] have the problem that the specific sensory information required to solve the task may have been suppressed by nonlinearities in the early sensory area, and no learning in the subsequent read-out connections could recover this information. Although these models could explain the task-specificity of perceptual learning by switching the read-out populations for different tasks [7,17], it remains unclear how such a switch should be implemented in neuronal terms. One option would be that the cognitive representation of the task in a higher cortical area would gate the activity to the appropriate read-out population while suppressing the other inappropriate read-out units. However, such a gating would again involve top-down projections, and it appears to be simpler to directly modulate the early stimulus representation by such a top-down signal.

Besides solving the task-switching problem, a task-dependent top-down modulation might also explain the longevity of perceptual learning which is not disturbed by repeated practicing of other tasks (see [16] and the review [29]). The top-down modulation is also consistent with the observation that perceptual learning in monkeys did neither change the receptive field size [30] nor the orientation tuning in V1 [31] during the performance of the trained task.

### Acquiring Top-Down and Bottom-Up Templates

We assume that a cognitive understanding of the task implies the selection of a task population in a higher cortical area with appropriate top-down projections to V1. Similarly, we assume that a decision population in a higher cortical area is selected which is driven by appropriate projections from V1. Such a pre-wiring must exist because no feedback from the external world about the performance in the perceptual task is given which may first shape the required synaptic connectivity. The synaptic top-down template encompasses the drive of the inhibitory populations together with the drive to the apical trees of the pyramidal neurons in V1. The bottom-up template selects a read-out network in some higher cortical area with an appropriate weighting of the feed-forward input. Both selection processes could themselves emerge from experience-dependent synaptic modifications during development [32] or during learning. For instance, it is conceivable that during the exposure to similar tasks, certain synaptic templates emerged based on intrinsic reinforcement signals or on a Hebbian type of synaptic plasticity [15,16]. These templates might be acquired subconsciously or even without explicitly performing a task [33].

### Attention and Time Course of Learning

The top-down modulation of the intrinsic V1 circuit during a task allows attention to operate through the same top-down template. In our model, the task neuron projecting down to V1 is directly driven by attention, making attention itself task-specific (Figure 3B1). Without external feedback, perceptual improvement is only possible in the case of weak, distributed attention (Figure 4B). Since learning in this case consists of strengthening the top-down template, it can be mimicked by increasing the attentional drive. In fact, the performance for distributed attention after learning reached the same level as for focal attention before learning. Learning with focal attention is not further possible because the common top-down pathways saturate. However, additional feedback on the correctness of the response may further lead to a fast reduction in the decision bias by modifying the readout synapses targeting the decision center (compare Figure 6A with Figure 4A1). In general, the fast initial progress often seen in perceptual learning [29] may reflect the adjustment of bottom-up connections to higher cortical areas, while the slow components of learning may follow the adjustment of top-down connections.

### Universal Top-Down Interactions

We hypothesize that perceptual learning is always accompanied by a top-down modulation of the lower sensory area. The top-down input may act in a twofold manner on the sensory area: (i) it may suppress (or enhance) the lateral connectivity by driving inhibition and (ii) it may modulate the gain of the pyramidal neurons. In functional terms, these top-down templates will (i) “de-contextify” (or “contextify”) the stimulus representation to suppress (or enhance) the perceptual bias and (ii) sharpen the stimulus representation to improve the discrimination sensitivity. Depending on the task, the two ingredients may be of different importance. If perceptual learning mainly consists in lowering some discrimination threshold such as in hyper-acuity tasks [16], a top-down gain increase may be enough. If perceptual learning includes the suppression or enhancement of intrinsic nonlinearities such as in context-enabled contrast discrimination [8,34] or in a bisection task [35], the modulation of the intrinsic circuitry will become crucial. Recent research has started to uncover these top-down templates [22,32,36], similarly to the uncovering of the bottom-up templates in terms of the neuron's (bottom-up) receptive fields.

### Multiple Use of a Canonical Microcircuitry

To implement the suppression of the flanking bar, we made use of a population of electrically coupled interneurons which inhibits a group of pyramidal neurons and receives feedback from these. Such a negative feedback circuitry represents a universal building block of the neocortex [19–21]. The same global inhibition can also enable competition among the pyramidal cells when operating in a high gain regime. This competition may enable winner-take-all behavior as it is used for decision making [27]. In fact, our decision network in the higher cortical area could be implemented by a similar local microcircuit used to linearize V1 and consisting of two (self-)excitatory populations which are both recurrently connected through the same inhibitory population (see Figure 2B1). As we were showing, the same canonical microcircuitry can be modulated to yield the suppression of brightness facilitation.

### Model Assumptions and Local Architecture

While the top-down recruitment of recurrent inhibition is one way to suppress lateral excitation, other local architectures in V1 yielding the desired suppression are also conceivable. The psychophysical phenomena alone are not constraining enough to postulate a unique neuronal implementation of the suppression effect. To make our additional model assumptions transparent, we recall the three psychophysical results the model explains. (1) Repeated practicing can reduce the facilitation in brightness discrimination induced by a flanking bar. (2) Focal attention without practicing can equally reduce facilitation, but the effects of perceptual training and focal attention do not add up when they are combined. (3) Similarly, repeated practicing and focal attention each may decrease the brightness discrimination threshold, but their combination does not lead to a further decrease.

An explanation of these phenomena requires at least three neuronal mechanisms operating on the early sensory area. (1) To account for the cancellation of lateral excitation during learning, we need to postulate some specific inhibition which is instantiated by the learning process. (2) Because focal top-down attention is equally effective in canceling lateral excitation as slow perceptual learning is, we also postulate a direct top-down recruitment of this inhibition. (3) Since the reduction in the brightness discrimination threshold is equivalent to an increase in the signal-to-noise ratio, we postulate a multiplicative enhancement of the sensory signal in the early representation. Again, because the threshold reduction can be induced by attention, the multiplicative modulation must be governed by a higher cortical center. Hence, the top-down recruitment of inhibition in V1 together with the top-down modulation of the neuronal gain represent the minimal number of assumptions which can account for the psychophysical data. Our two additional assumptions that the top-down drive should roughly match the threshold for inhibition, and that the synaptic strength of the feedback inhibition should roughly match the synaptic strength of the lateral excitation, are a consequence of explaining the suppression effect by means of recurrent inhibition. Other ways of implementing the top-down suppression may not need these additional assumptions. For a generalization of the suppression mechanism to multiple neurons and for alternative wirings, see below and Figure S1.

### Alternative Local Microcircuits

As a first alternative explaining the top-down suppression, we may consider the scenario of non-recurrent lateral inhibition. Each excitatory neuron which laterally projects to a target neuron is postulated to also project through an inhibitory companion neuron onto the same target neuron. Without top-down input, the companion neuron is silent, but in the presence of a top-down depolarization it inhibits the target neuron as strongly as this is excited, effectively canceling the lateral excitation. Besides being highly specific, such a wiring suffers from the same problem of fine-tuning (see Figure S1).

As a second alternative, all excitatory lateral connections onto the target neuron might first be funneled through a specific population of excitatory neurons before they effectively excite the target neuron. This additional population just has to linearly feed through the excitatory input. But top-down input can now easily inhibit this population and cut off any lateral excitation, without affecting the activity of the source neurons. Although this version would require less tuning of inhibition, it makes an even stronger assumption on the lateral excitatory wiring. One advantage of the non-recurrent inhibition, though, is that it would not require the additional top-down gain increase at the intermediate layer 5 neurons (Figure 3B1).

In reality, the different local suppression mechanisms discussed above might act in parallel. Whatever the specific implementation is, the top-down modulation of the suppression mechanism(s) remains an appealing paradigm to explain the reduction in brightness facilitation with perceptual training. The fact that top-down input may operate in different ways to achieve the same result reflects the generality and flexibility of this concept.

Alternative mechanisms exist also to implement the top-down gain modulation, here required to explain the reduction in the brightness-discrimination threshold. In addition to the suggested dendritic calcium currents [24], other mechanisms on the level of a single neuron [37–39] or of a recurrent network [40] are conceivable which may yield an appropriate top-down gain modulation.

### Experimental Predictions

The suggested mechanisms underlying the suppression of the perception bias and the reduction of the discrimination threshold would permit a specific pharmacological modulation of perceptual learning (see also [41]).

(1) Any experimental manipulation which would modulate the top-down input would have behavioral implications. For instance, blocking GABA receptors in vivo in monkeys by local perfusion or in humans by medications would prevent the top-down suppression. After unsupervised practicing, the brightness facilitation for both attentional states would then be still as high as the original facilitation for distributed attention before practicing.

(2) A more specific test of the model would be to activate GABA_B_ receptors by Baclofen to prevent the gain increase of L5 pyramidal neurons [42,43]. As a consequence, the discrimination threshold would remain high (Figure 6B), while brightness facilitation may still get suppressed. An interesting option is to lower the excitability of human V1 by repetitive transcranial magnetic stimulation (rTMS) [44]. rTMS may recruit inhibition and block the gain increase through GABA_A_ and GABA_B_ receptor activation. Again, this is expected to increase the discrimination threshold while the facilitation may still get suppressed.

(3) Finally, learning in the presence of an external feedback may help to disentangle the contribution of bottom-up and top-down inputs. A teacher feedback may lead to a complete suppression of the facilitation by the flanking bar (Figure 6A). Such a further reduction of the perception bias would be consistent with the effect of the teacher feedback in context-dependent orientation discrimination [17]. However, although the discrimination threshold was further reduced by a teacher feedback in a Vernier task [45], it was not reduced in the orientation discrimination task [17] nor in our model for brightness discrimination (simulations, unpublished data). In the model, the differential effect of the teacher feedback on the perception bias and the discrimination threshold arises because the teacher signal is assumed to only affect learning in the decision circuitry of the higher cortical area, and not the representation network within the lower sensory area.

## Materials and Methods

### Model stimuli.

To account for Weber's law stating that perception scales logarithmically with the stimulus intensity, the inputs x_i_ into V1 encoding the test, flank and reference bar are chosen to be logarithmic functions of the stimulus brightness, x_i_ = 35log(L_i_ + 1.5), where L_i_ (i = 1,2,3) denotes the luminance of the test, flank, and reference bar, respectively. The luminance values are set to match the luminance ratios of test bar and the flank bar to the reference bar used in the experiments [1,18]. The reference bar luminance is fixed to L_ref_ ≡ L_3_ =4, and the test bar luminance is one out of the seven different brightness levels L_test_ ≡ L_1_ = 1,2,…,7 (arbitrary units). The luminance of the flanking bar is always slightly above the one of the test bar, L_flank_ = L_test_ + 0.05, as chosen in the brightness discrimination experiment (Figure 3A).

### Recurrent network dynamics.

The firing rates of the prototypical excitatory L2/3 neurons (each representing homogeneous neuronal population) are characterized by

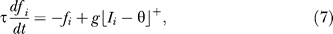
with [z]^+^ = max(0,z), a time constant τ = 20 ms, and a gain g which is a monotonically increasing function of the top-down firing rate f_task_ as described below. The prototypical L2/3 pyramidal neuron encoding the test bar (i = 1) and flank bar (i = 2), respectively, receives the total input current I_i_ = x_i_ +w_ij_f_j_ + λf_task_ − kf_inh_ (i,j ∈ {1,2}, i ≠ j), where x_i_ is the feed-forward input, w_ij_ the lateral synaptic strength, λ = 0.2 the dendritic attenuation factor for the top-down input projecting to the distal dendrite, and k the strength from the lateral inhibition.

The dynamics of the two prototypical inhibitory neurons are defined by

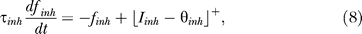
with a time constant τ_inh_ = 5ms (for other parameter values see the caption of Figure 1). For the inhibitory neuron which is related to the excitatory L2/3 neurons representing the test and flanking bar, the total input current is given by I_inh_ = f_1_ + f_2_ + w_task_ 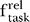 (Figure 3B1). Here, w_task_ denotes the synaptic strength of the top-down input to the inhibitory neurons (Figure 3B1) and 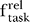 is the synaptic release rate undergoing short-term depression (see Figure 1 and the definition below). Setting 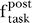 = w_task_ 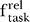 and df_inh_/dt = 0 in Equation 8 yields the steady state firing rate f_inh_ = [f_1_ + f_2_ + 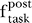 − θ_inh_]^+^ (cf. also Equation 2 in the main text).

The firing rate of the L2/3 neuron which encodes the reference stimulus (f_3_ ≡ f_ref_) is governed by the dynamics (Equation 7) with input current I_3_ = x_3_ − kf_inh_. The corresponding inhibitory neuron is again governed by Equation 8, but with an input current I_inh_ = f_3_ + w_task_ 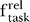 , i.e., with f_1_ + f_2_ replaced by f_3_ (with i = 1,2,3 standing for “test”, “flank”, and “ref”, respectively).

Finally, the task neuron is driven by an attentional neuron with a firing rate f_att_. This attentional input is weak in the case of distributed attention, 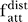 = 16 Hz, and strong in the case of focal attention, 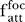 = 48 Hz. The firing rate of the task neuron is proportional to the attentional input, f_task_ = w_att_f_att_, with w_att_ being the synaptic strength from the attentional center to the task neuron. This top-down weight w_att_ undergoes slow Hebbian modifications (see Equation 14 below).

### Top-down gain modulation.

The top-down input from the task neuron to V1 changes the gain of the L2/3 and L5 pyramidal neurons. The gain g of the L2/3 neurons increases with f_task_ according to

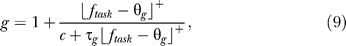
with c = 2.4, θ_g_ = 8.5 Hz, and τ_g_ = 0.8s.

L5 pyramidal neurons receive a single bottom-up somatic input from their co-aligned L2/3 neurons and a top-down dendritic input from the task neuron. The overall somatic current to a L5 neuron is Ĩ_i_ = f_i_ + λf_task_ (i = 1,2,3), where λ = 0.2 is the dendritic attenuation factor and i = 1,2,3 standing for “test”, “flank”, and “ref”, respectively.

The firing rate of the L5 neurons is determined by

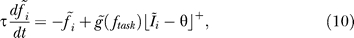
with the same time constant τ and threshold θ as for the L2/3 pyramidal neurons (i as above). Similarly, the gain gx̃ monotonically increases with f_task_ according to the same right-hand side of Equation 9, but with the parameter values c = 1.2 and τ_g_ = 0.4s and θ_g_ = 8.5Hz. This parameter choice leads to a gain function which is twice as steep and saturates at twice the level of the corresponding function for L2/3 pyramidal cells (M. Larkum, unpublished data, see also [24])

### Short-term synaptic depression.

We introduced synaptic short-term depression in the top-down projection to the inhibitory neurons (Figure 2B1, inset). The synaptic release rate at these connections is given by the product of the release probability and the presynaptic firing rate, 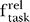 = p_rel_f_task_. The release probability itself is a dynamic variable and is proportional to the vesicle recovery probability, p_rel_ = up_rec_, with proportionality constant u interpreted as a fraction of transmitter use per release. The dynamics of the vesicle recovery probability is given by

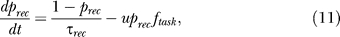
where τ_rec_ is the vesicle recovery time constant, see [23] or [46]. We set u = 0.4 and τ_rec_ = 0.1s. In the steady state the release rate becomes

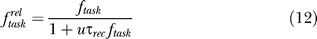
and it is reached with an effective time constant τ_rec_/(1 + uτ_rec_f_task_). For presynaptic frequencies f_task_ beyond the critical input frequency f_crit_ = 1/(uτ_rec_) (=25 Hz), the release rate 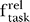 saturates at the same value (of 25 Hz).

Synaptic depression in the top-down connection to the inhibitory neurons is introduced to achieve a constant drive at high top-down frequencies. According to Equation 8 and its subsequent remarks, the steady state firing rate for the recurrent inhibition among the test and flanking neurons in layer 2/3 is

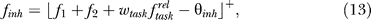
see also Equation 2. To obtain the linearized firing rate in Equation 3, f_inh_ = f_1_ + f_2_, the effective top-down input w_task_ 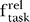 must roughly match the firing threshold θ_inh_. This requires that the postsynaptic current generated by the top-down input remains approximately constant. Such a constant drive is in fact achieved by synaptic short-term depression for f_task_ ≫ f_crit_, as expressed by Equation 12. To obtain the match w_task_ 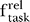 ≈ θ_inh_, for large presynaptic firing rates we set w_task_ = 1.9 θ_inh_uτ_rec_.

### Stimulation protocol and decision making.

To take account of the temporal processing during the experiment underlying Figure 4, we run the network with the same schedule for the stimulus presentation as in the experiments [1,18]. For each presentation, the attentional condition (focal/distributed), the contextual condition (flank/no flank), and the luminance of the test bar (L_i_, see above) are randomly chosen.

A virtual “attentional cue” (Figure 3A1) turns on top-down input from the attentional center to the task center, 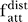 or 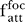 (Figure 3B1), representing either “distributed” or “focal” attention and remains active throughout the stimulation protocol up to the final decision. 1.5 s after the attentional onset, the stimulus is flashed for 0.1 s. Each stimulus consists of a reference bar and a test bar with or without flank. The decision about the brightness difference between test and reference bar is drawn 0.9 s after stimulus offset based on the activities of the L5 pyramidal neurons at that time. In the case of unsupervised learning (i.e., without feedback), the total postsynaptic current entering in the decision function is given by I_dec_ = fx̃_test_ − fx̃_ref_ (Figure 3A2).

To mimic noisy neuronal decision making [27], the decision y_dec_ = 1 (test bar judged to be brighter than reference bar) is chosen with probability p = p(I_dec_) = 0.5(1 + erf(I_dec_/d)), and the decision y_dec_ = 0 with probability 1 − p, where d = 2/15 and erf(x) is the standard error function. After the decision making, the neuronal firing rates are reset to 0 and short-term synaptic depression is put to the recovered state p_rec_ = 1. A “trial” consists of three (in the experiment it was one to six) stochastically independent stimulus presentations including decision making.

### Unsupervised perceptual learning.

To compare with the experiment [1,18], we trained our model network with 600 (in the experiment it was 500–800) trials per “week”. During the stimulation protocol, the strength of the attention-to-task synapse w_att_ (Figure 3B1) changes according to the Hebbian rule

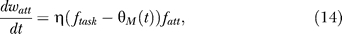
with a factor η = 3·10^−8^ s, an initial value w_att_ = 0.5, and hard bounds for w_att_ at 0 and 1. The modification threshold θ_M_(t) is itself slowly following the postsynaptic firing rate f_task_ according to

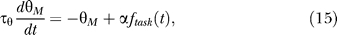
with an adaptation time constant τ_θ_ = 60s, a proportionality constant α = 0.8, and an initial value of θ_M_ = 10Hz. In the unsupervised learning scenario, w_att_ steadily increased until it reached the upper bound 1 after roughly 10,000 trials (17 weeks).

All differential equations were integrated with forward-Euler using a time step of dt = 0.3 ms.

### Perceptual learning with feedback.

In the case of supervised learning (as underlying Figure 6A), the current entering in the decision network is given by I_dec_ = wx̃_test_fx̃_test_ + wx̃_flank_fx̃_flank_ + wx̃_ref_fx̃_ref_, where the wx̃_i_ reflect the weights emerging from the L5 pyramidal neurons (Figure 3B). In addition to the top-down weight w_att_ (Equations 14 and 15), we modified the bottom-up weights wx̃_i_ (with i = 1, 2, and 3 standing for “test”, “flank”, and “ref”, respectively) according to the perceptron learning rule [47]: whenever the output of the decision unit was correct, no modification of the wx̃_i_ was made, while otherwise the synapses change in an anti-Hebbian way.

Formally, we consider a reward signal R with R = 1 if the network decision y_dec_ is correct, and R = 0 otherwise (where “correct” means that y_dec_ = 1 if the test bar is brighter than the reference bar, L_test_ > L_ref_, and y_dec_ = 0 if the test bar is equal or less bright than the reference bar, L_test_ ≤ L_ref_). The synaptic strength *wx̃_i_* is then changed according to

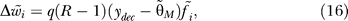
with learning rate q = 0.0001, a modification threshold θ̃_M_ = 0.5, and i = 1, 2, and 3 standing for “test”, “flank”, and “ref”, respectively.

Because we assume that the choice of the decision network is appropriate for an unbiased discrimination, we choose initial weights wx̃_test_ = 0.5, wx̃_flank_ = 0.0, and wx̃_ref_ = −0.5. The average reward R increases throughout the simulations from 0.5 towards roughly 0.9. To enhance the undershoot of the learning curve for focal attention, only 15% of the presentations were with focal attention and 85% with distributed attention. During the learning procedure, the top-down weight w_att_ is subject to the dynamics in Equation 14 and Equation 15 and steadily increases to a value of 0.74 at the end of the learning process (12,000 trials).

### Analysis of the model data.

The brightness facilitation (shift in brightness perception of the test bar induced by the presence of the flanking bar) and the discrimination threshold (just distinguishable brightness difference relative to the absolute brightness) were extracted from the psychometric curves described below using the *probit* method (cf. [1] and [48]). After each block of 600 trials, stimuli presented under the same attentional and contextual conditions (focal/distributed, flank/no flank) and the same brightness were pooled together. For each pair of attentional and contextual condition, the ratio of positive responses to a particular test luminance, L_test_, was plotted against the logarithm of the relative luminance, log(L_test_/L_ref_). The four sets of 2-D data points were fitted by a “normal cumulative distribution function” (approximating the decision probability *p* as a function of the logarithmic relative luminance), yielding the psychometric response curves for the different conditions. Facilitation was identified by the left shift (at the 50% correctness level) of the fit for the flank relative to the non-flank condition. The detection threshold was identified by the maximal slope of the fitting curve for the no-flank condition.

## Supporting Information

Figure S1 Different Wirings for Suppressing Lateral Excitation (A) Recurrent inhibition (version investigated in the paper). A few inhibitory neurons (filled circles) which are recurrently connected to a population of excitatory principal neurons (open circles) with receptive fields aligned in a row (black dots represent excitatory synapses, diamonds inhibitory synapses). The principal neurons which laterally excite each other will also inhibit each other through the corresponding inhibitory neuron, provided that top-down input (not shown) drives this inhibitory neuron towards threshold.
(B) Unidirectional inhibition (alternative 1). Each principal neuron may have its own inhibitory neuron which is driven by the surrounded principal neurons. Lateral excitation is canceled by properly tuning the inhibitory loop running in parallel to the lateral excitation. As in (A), the strengths of the inhibitory loop and the top-down input have to be fine-tuned.
(C) Funneling lateral excitation (alternative 2). If lateral excitation would drive the principal neurons through individual bottleneck-neurons (small open circles), it would be easy to suppress the excitation by inhibiting these bottlenecks. Although no fine-tuning is required in this solution, a fairly specific connectivity pattern among the excitatory neurons is assumed.
(34 KB PDF)

## Abbreviations

LTP: long-term potentiation
V1: primary visual cortex
