# Supplementary material for: Perceptual Learning via Modification of Cortical Top-Down Signals
Source: PLoS Comput Biol. 2007 Aug 17;3(8):e165. doi: 10.1371/journal.pcbi.0030165 (PMC1950342; doi:10.1371/journal.pcbi.0030165)
Supplement: Supplementary file 1 [file pcbi.0030165.sg001.pdf]

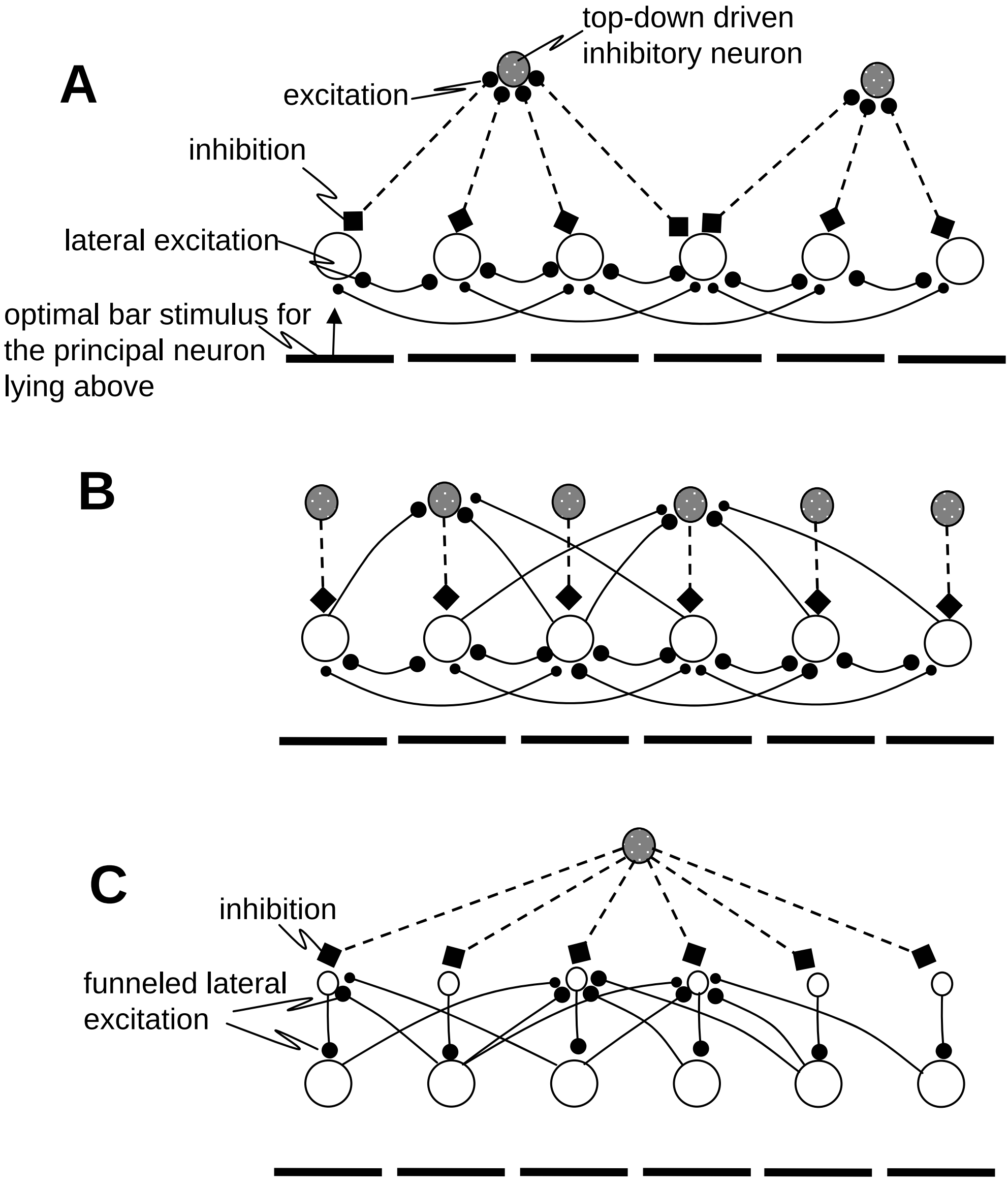
A
top-down driven
inhibitory neuron
excitation
inhibition
lateral excitation
optimal bar stimulus for
the principal neuron
lying above
B
C
inhibition
funneled lateral
excitation
